# Genomic surveillance inequities in emerging infectious diseases: Why fragile settings like Somalia are critical to global health security

**DOI:** 10.1016/j.nmni.2026.101752

**Published:** 2026-04-09

**Authors:** Abdikadir Ahmed Hassan, Yakub Burhan Abdullahi, Liibaan Abdulahi Sudi, Mohamed Daud Mohamed, Iqro Omar Ibrahim

**Affiliations:** a Faculty of Health Sciences and Tropical Medicine, Somali National University, Mogadishu, Somalia; b Faculty of Health Sciences, Salaam University, Mogadishu, Somalia

Dear Editor,

The rapid expansion of SARS-CoV-2 genomic sequencing during the COVID-19 pandemic underscored the transformative role of pathogen genomics in real-time variant tracking, vaccine development, and evidence-informed public health decision making [1]. However, this progress has been uneven, exposing a structural inequity: the infrastructure underpinning genomic surveillance remains disproportionately concentrated in high-income settings, leaving large regions effectively absent from global pathogen monitoring systems [1].

Global analyses have demonstrated the extent of this imbalance. During the first two years of the pandemic, 78% of high-income countries sequenced more than 0.5% of confirmed cases, compared with only 42% of low- and middle-income countries, with markedly slower data turnaround times in the latter group [1]. Despite bearing a substantial disease burden, Africa contributed approximately 1% of the globally shared sequences [2]. Such disparities are not merely reflective of resource constraints; they introduce systematic distortions into the global epidemiological evidence base that informs surveillance strategies, risk assessments, and response planning [1,2].

Somalia exemplifies this exclusion with clarity. As a fragile and conflict-affected state characterized by protracted instability, large-scale internal displacement, and severe workforce shortages, the country lacks functional genomic sequencing capacity [3]. Its International Health Regulations compliance score remains low, reflecting broader systemic limitations in surveillance, laboratory infrastructure, and response capacity [4]. Although incremental progress has been made through integrated disease surveillance initiatives, the transition from syndromic reporting to molecular characterization of pathogens has not yet been realized. Consequently, Somalia has not contributed consistent genomic data to regional or global platforms, rendering its epidemiological dynamics effectively invisible [3,4].

The implications of such exclusion extend beyond the national boundaries. Settings that are absent from genomic surveillance networks may serve as reservoirs for undetected transmission and potential variant emergence, delaying the recognition of evolving threats. Moreover, the absence of data from fragile contexts introduces bias into global datasets, limiting the generalizability of pathogen evolution models and potentially skewing priorities for vaccine development, therapeutic targeting, and resource allocation [1,2]. In this sense, genomic surveillance inequity is not only a matter of access but also a fundamental limitation of the integrity of global health intelligence.

Addressing this gap requires a shift from technology-centric expansion to an equity-oriented system design. Regional genomic hubs, as advanced through continental initiatives, offer a pragmatic pathway to extend sequencing access to countries that lack standalone capacity [5]. Complementary strategies, including portable sequencing technologies, cloud-based bioinformatics, targeted workforce development, and structured technology transfer, are essential for enabling sustainable integration.Equally important are equitable data governance frameworks that balance timely global sharing with national ownership and trust [5].

Global health security is inherently collective and contingent on the inclusiveness of surveillance systems. As long as fragile settings such as Somalia remain excluded from genomic surveillance networks, the global architecture for detecting and responding to emerging infectious diseases will remain inadequate. Therefore, integrating these contexts is not peripheral to preparedness but central to it.

## CRediT authorship contribution statement

**Abdikadir Ahmed Hassan:** Conceptualization. **Yakub Burhan Abdullahi:** Supervision. **Liibaan Abdulahi Sudi:** Methodology. **Mohamed Daud Mohamed:** Writing – review & editing. **Iqro Omar Ibrahim:** Investigation.

## Declaration of competing interest

The authors declare that they have no known competing financial interests or personal relationships that could have appeared to influence the work reported in this paper.
